# ASYM: multimodal depression recognition via mamba-enhanced attentive feature fusion

**DOI:** 10.3389/fpsyt.2026.1704005

**Published:** 2026-04-13

**Authors:** Caijian Hua, Zhuofu Wen, Liuying Li, Xia Zhou

**Affiliations:** 1 School of Computer Science and Engineering, Sichuan University of Science and Engineering, Yibin, China; 2 Traditional Chinese Medicine Department, Zigong First People’s Hospital, Zigong, China

**Keywords:** cross-modal attention, depression detection, feature pyramid network, mamba, multi-modal feature fusion

## Abstract

**Introduction:**

Depression is a prevalent mental disorder with a severe global impact. Traditional interview-based assessments are limited by subjectivity, lengthy procedures, and unequal access to care. Although advances in AI have facilitated multimodal models for depression detection—using audiovisual data as an accessible alternative to biosignals—current approaches remain challenged by inefficient long-term temporal modeling and superficial multimodal fusion. Moreover, biosignal-based methods are constrained by high costs and narrow applicability. These challenges underscore the urgent need for optimized multimodal solutions.

**Methods:**

This paper proposes ASYM (Attentive Synergy Mamba), a novel multimodal architecture for depression recognition, comprising three core modules: a Cross-Modal Interactive Mamba, a Multi-Scale Gated Parallel Fusion, and a Multimodal Enhanced Mamba. First, features from each modality are interactively enhanced using convolutional neural network and Bi-Mamba blocks. Cross-modal complementary information is then extracted via a cross-attention mechanism. A dual-path fusion module subsequently augments multi-scale representations and integrates cross-modal features through dynamic weighting. Finally, the feature representations are refined by a series of Bi-Mamba blocks.

**Results:**

Evaluations on the D-Vlog and LMVD datasets using accuracy, precision, recall, and F1-score showed that ASYM achieved an accuracy of 70.91% and an F1-score of 77.13% on D-Vlog, and 74.68% accuracy with a 74.90% F1-score on LMVD. The macro-average performance across both datasets surpassed all compared mainstream methods. Ablation studies confirmed the necessity of each component, as removing any module significantly degraded performance, underscoring the efficacy and critical contribution of the proposed architecture.

**Discussion:**

While multimodal depression detection has improved upon single-modality approaches, issues such as computational inefficiency in long-sequence processing and inadequate fusion strategies persist. Our model addresses these limitations through multimodal interaction and multi-scale feature fusion. Future work will focus on clinical validation across diverse populations to bridge computational psychiatry and clinical practice.

## 1 Introduction

Major Depressive Disorder (MDD), the leading cause of global disability, affects over 320 million individuals and contributes to more than 800,000 annual suicide deaths (1). Conventional diagnostic approaches reliant on clinical interviews and scale assessments (e.g., CES-D, PHQ-9) exhibit inherent limitations, including subjectivity, temporal inefficiency, and uneven healthcare resource allocation, thereby complicating early screening and dynamic monitoring (2–6). Recent advancements in artificial 35 intelligence (AI) have catalyzed the development of multimodal machine learning (ML) models for depression detection, offering novel pathways to enhance diagnostic objectivity and mitigate misdiagnosis.

Recent progress in biosignal-based detection methodologies has demonstrated significant success. Electroencephalography (EEG) and magnetic resonance imaging (MRI) techniques leverage neural activity features to classify depressive states. While these models exhibit considerable promise in accurate identification of depression, their clinical utility remains constrained by prohibitive equipment costs and limited application scenarios (7–10). These limitations have motivated research into low-cost, accessible multimodal data fusion strategies.

In this work, we propose a novel Attentive Synergy Mamba Network (ASYM) for interactive enhancement and fusion of cross-modal time-series features, aiming to classify depression-specific content. The network integrates a cross-modal attention mechanism that first refines features from each modality via CNN and Bi-Mamba blocks, then establishes fine-grained correlations to capture complementary intermodal information. Furthermore, a dual-path fusion module is designed: the multimodal feature pyramid hierarchically aggregates multi-scale contextual representations, while the adaptive fusion submodule achieves comprehensive cross-modal integration via dynamic weight allocation. Finally, Bi-Mamba blocks further enhance fused features. Our contributions are summarized as follows:

- A novel multimodal fusion architecture that ameliorates temporal misalignment through bidirectional feature-sequence interaction.
- A bidirectional cross-modal attention module that specifically mitigates inter-modal noise and behavioral degradation phenomena via multi-scale aggregation.
- State-of-the-art accuracy on D-Vlog and LMVD datasets, outperforming existing methods.

## 2 Related work

This section presents a concise overview of contemporary research in depression detection, Mamba architectures, and multimodal multi-scale fusion strategies.

### 2.1 Depression detection

Recent advances in deep learning have substantially propelled Major Depressive Disorder (MDD) recognition through both unimodal and multimodal approaches. Li et al. (2024) introduced the SFTNet model (11), demonstrating facial micro-expression analysis as a viable auxiliary diagnostic tool through spatio-temporal variation analysis of controlled emotional responses. Concurrently, Zhang et al. (2025) proposed DepITCM (12), establishing that hierarchical audio-visual fusion with multi-task learning enhances detection accuracy. Zhou et al. (2025) proposed an Audio Modality Encoder (AME) and a Visual feature fusion Module (VCM), which enhance the expressiveness of visual cues by integrating audio features with facial visual representations (13). Liu et al. (2024) developed the LGMF-GNN system (14), a local-to-global multimodal graph neural network that quantifies pathology and generates multi-scale evidence for precision treatment. Complementarily, Zhang et al. (2024) introduced an ensemble voting model (15) integrating textual, audio, and video features, outperforming unimodal baselines. Zhou et al. (2025) proposed the STCM-Mamba framework, which achieves effective integration of spatio-temporal features and strikes a balance between efficient long-sequence modeling and computational complexity (16). Zhou et al. (2026) proposed CAF-Mamba, a Mamba-based cross-modal adaptive attention fusion framework that overcomes limitations of insufficient features and naive static fusion, and enables effective multimodal fusion by capturing explicit and implicit cross-modal interactions while dynamically weighting each modality (17). Collectively, these studies reflect an emerging paradigm shift toward multimodal collaboration and clinical translation.

### 2.2 Mamba and state space models

Gu and Dao (2023) pioneered the Mamba architecture to resolve computational and memory bottlenecks in Transformer-based long-sequence processing (18). As a state space model, Mamba enhances long-sequence efficiency through parameterized state transitions and update mechanisms, enabling superior long-range dependency capture. Compared to Transformers, it offers significant advantages in computational efficiency and flexibility for large-scale sequences, substantially reducing resource overhead (18, 19). Consequently, Mamba serves as an optimal lightweight solution for Vlog-based depression detection, balancing efficiency and cost-effectiveness.

### 2.3 Multimodal multi-scale fusion

Multimodal multi-scale fusion integrates heterogeneous data (e.g., visual, auditory, textual) across spatial and temporal scales to enhance complex information understanding. The Feature Pyramid Network (FPN) exemplifies this approach (20), hierarchically fusing features to balance semantics and spatial details while improving scale-invariant robustness. Despite its medical applicability, standard FPN exhibits semantic misalignment in Vlog-based depression detection. To address this, we integrate Gated Dynamic Fusion (21, 22), which suppresses conflicting features and reinforces cross-modal semantic consistency.

## 3 Materials and methods

### 3.1 Datasets and preprocessing

This study employs two benchmark multimodal datasets for depression detection: D-Vlog and LMVD (23, 24). These datasets facilitate cross-platform validation; LMVD captures Eastern and Western cultural nuances, while D-Vlog emphasizes spontaneous behavioral patterns, thereby supporting robust depression detection in uncontrolled environments. Furthermore, a temporal random cropping strategy was applied to the training sets of both datasets for data augmentation. Notably, the pre-extracted audio and visual features provided by these datasets were used in their original form, with no cross-dataset normalization or standardization applied.

#### 3.1.1 D-Vlog dataset

The D-Vlog dataset consists of 961 YouTube video blogs, totaling 160 hours of content. Depression labels for this dataset were generated through a manual, rule-based annotation process. Specifically, vlogs were labeled as depressed if the creator explicitly described current clinical symptoms (e.g., suicidal ideation), without reliance on questionnaire-based thresholds. The dataset focuses on non-verbal cues associated with depression and provides features including eGeMAPS acoustic descriptors and facial landmarks. In our experiments, the dataset was partitioned into training, validation, and test sets in a 7:1:2 ratio, as detailed in **Table 1**.

**Table 1:** Train/validation/test sample counts for the D-Vlog dataset.

Category	Train	Val	Test	All
Depression	389	55	111	555
Non-Depression	284	41	81	406
All	673	96	192	961

#### 3.1.2 LMVD dataset

The LMVD dataset, the largest publicly available dataset in this field, comprises 1,823 video blogs (214 hours) from 1,475 participants, collected from multiple platforms including Sina Weibo, Bilibili, TikTok, and YouTube. It features clinically validated depression labels. The annotation process involved multiple stages: an initial manual screening to filter vlogs based on quality, followed by verification by clinicians who aligned the final labels with DSM-V symptom criteria (e.g., agitation or slowed speech). Focusing on rich multimodal depression-related cues, the dataset provides features such as VGGish audio embeddings, facial action units (FAUs), and gaze dynamics. In our experiments, the dataset was partitioned into training, validation, and test sets in an 8:1:1 ratio, as detailed in **Table 2**.

**Table 2:** Train/validation/test sample counts for the LVMD dataset.

Category	Train	Val	Test	All
Depression	726	91	91	908
Non-Depression	732	91	92	915
All	1458	182	183	1823

#### 3.1.3 Data augmentation

**Figure 1:**
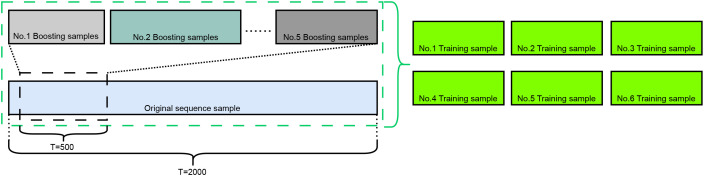
The random segment sampling method for sequence data augmentation.

For each original sample (a sequence with temporal length T and feature dimension F), we generated up to five augmented sub-sequences. This process is illustrated in **Figure 1**, which depicts the relationship between original sequences and their augmented counterparts. The generation procedure consists of three steps:

1. Randomly determining a fragment length *f*_length_ (with the constraint $f_{\text{length}}=500$) to ensure sufficient temporal information.
2. Randomly selecting a starting position *t*_start_ within the valid range of the original sequence.
3. Extracting the sub-sequence from *t*_start_ to *t*_start_ + *f*_length_ as a new augmented sample, which inherits the original sample’s label.

This augmentation strategy enriches the training data by introducing diverse temporal segments, thereby enhancing the model’s robustness to variations in sequence length and start position.

### 3.2 Method

**Figure 2:**
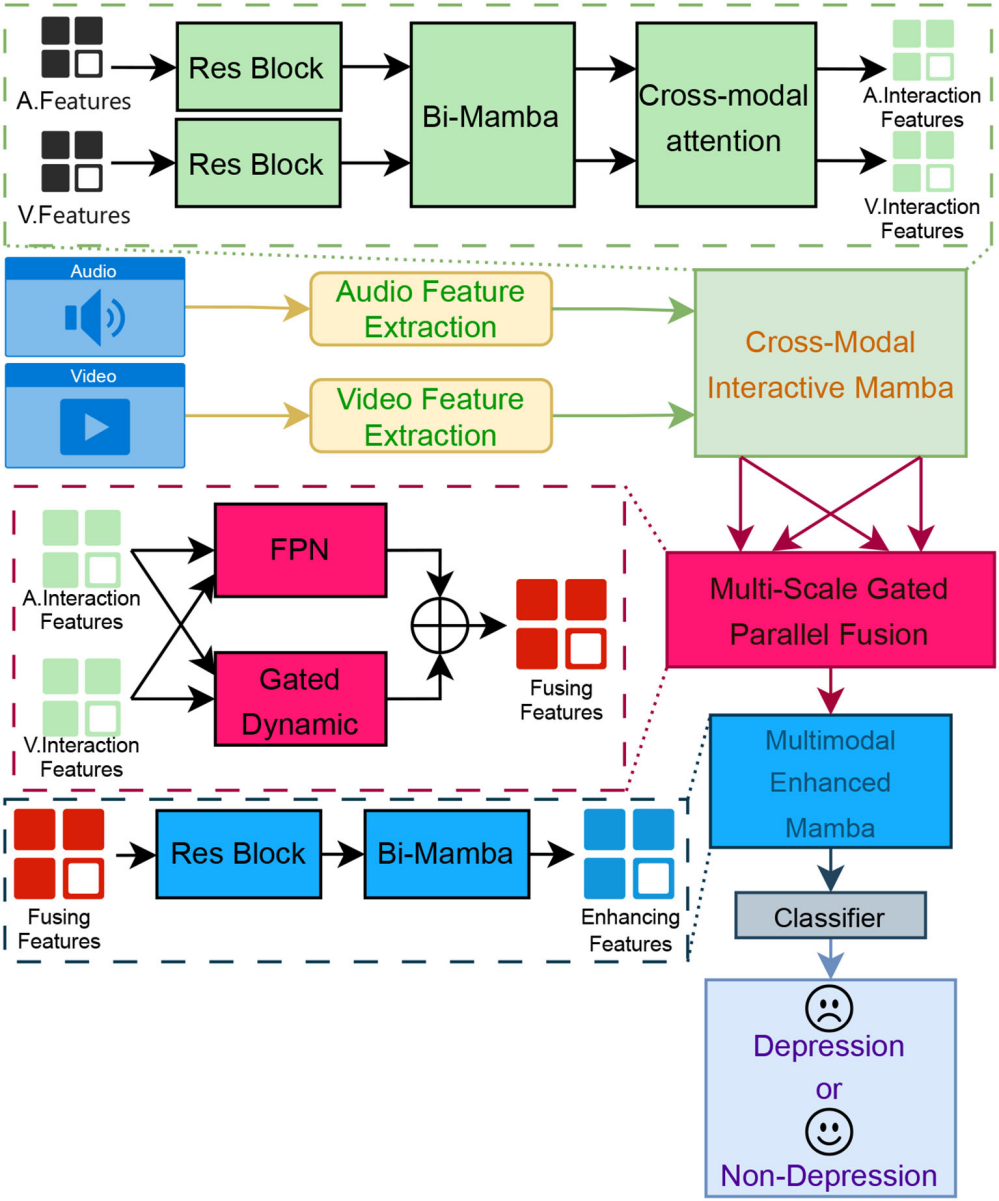
Overall architecture of the proposed multimodal depression recognition framework.

As illustrated in **Figure 2**, the proposed multimodal depression recognition framework consists of a threestage cascaded architecture: a CrossModal Interactive Mamba, a MultiScale Gated Parallel Fusion module, and a Multimodal Enhanced Mamba. This section provides a comprehensive exposition of the methodological underpinnings and operational workflow of this hierarchical architecture.

#### 3.2.1 Cross-modal interactive mamba

**Figure 3:**
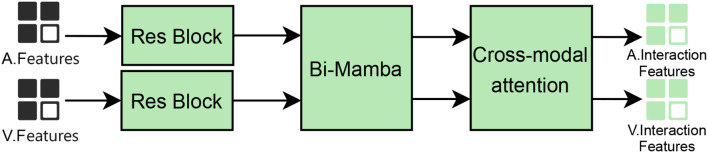
Cross-modal feature interaction process via bi-mamba and attention.

The structure of this module is shown in **Figure 3**. In this model, the dataset comprises video data, where each sample is decomposed into audio and video frame features.

**Figure 4:**
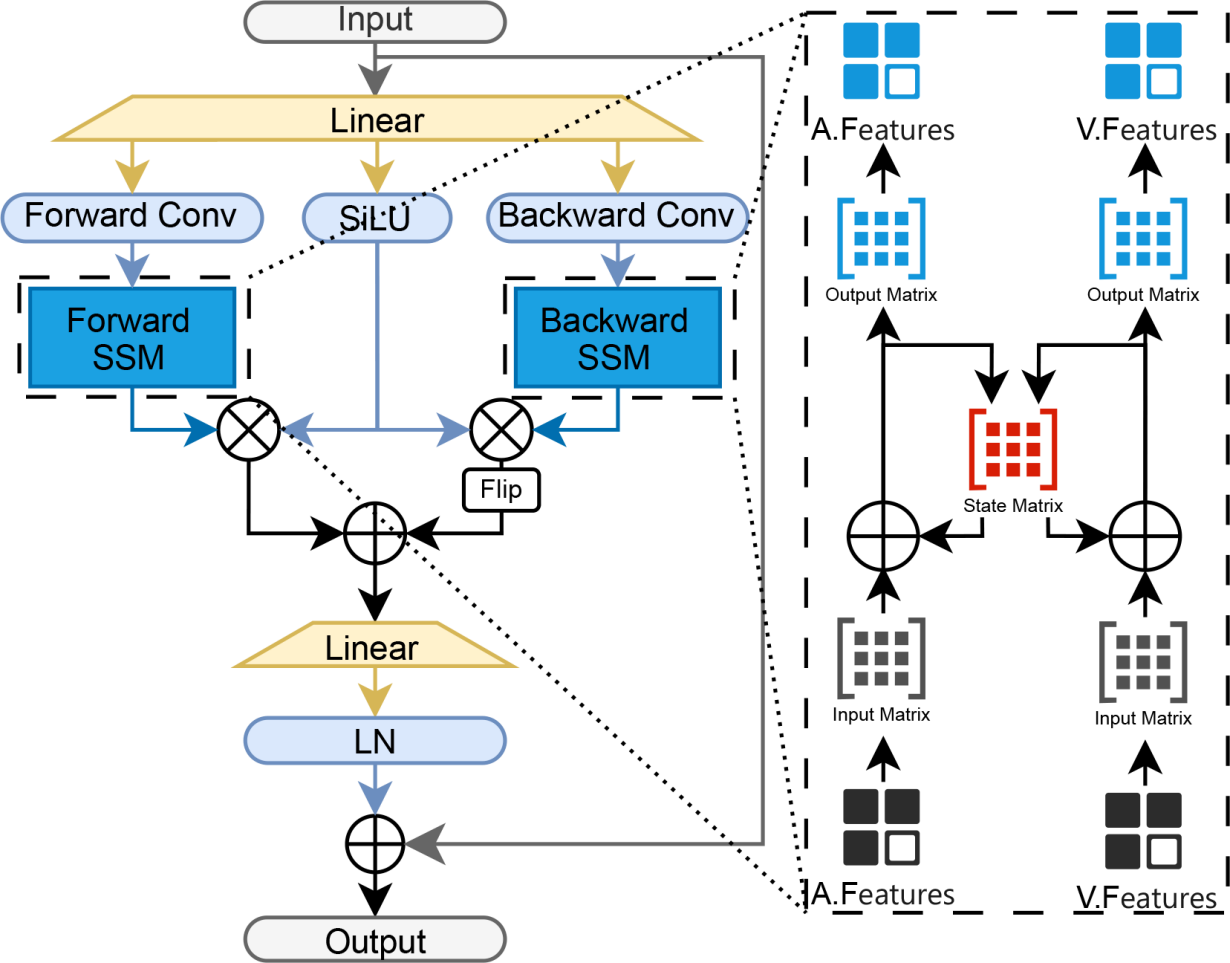
Detailed architecture of the bi-mamba module.

In the multimodal feature interaction stage, we first employ bidirectional Mamba as the baseline model to achieve preliminary interaction between features from different modalities, as illustrated in **Figure 4** (26). The processed input features are first projected through a linear layer. The resulting representation is then split into two parallel processing streams. In the forward stream, features pass sequentially through a one-dimensional convolution (Forward Conv), a SiLU activation, and a forward-oriented state-space model (Forward SSM) that captures temporal dependencies conditioned on past context. Simultaneously, the backward stream applies a one-dimensional convolution (Backward Conv) directly to the reversed feature sequence (from tail to head); its output is then flipped back to the original order, activated by SiLU, and modeled by a backward-oriented state-space model (Backward SSM) to incorporate future context.

A key design is that the forward and backward SSMs share the same state-transition matrix with their counterparts processing the other modality. This shared dynamic forces features from different modalities to evolve toward a common latent state representation during bidirectional processing, thereby laying a consistent foundation for cross-modal alignment (25).

Finally, the outputs of the forward SSM and the backward SSM (after flip-back) are concatenated, integrating both historical and future contexts. The fused representation is then passed through a final linear projection followed by layer normalization (LN), producing the interacted output features (25, 26).

To mathematically formalize this bidirectional temporal modeling paradigm, the state transition dynamics are derived through the following recursive formulation (Equations 1, 2):

(1) $$h_{t}^{\text{forward}}=\bar{A}h_{t-1}^{\text{forward}}+\bar{B}x_{t}$$

(2) $$h_{i}^{\text{backward}}={\bar{A}}_{b}h_{i+1}^{\text{backward}}+{\bar{B}}_{b}x_{t}$$

Here, *A* denotes the state transition matrix, *x_t_*represents the input at time step *t*, and *B* corresponds to the modality-specific input parameter. The corresponding output is computed calculated using the following formulation (Equations 3, 4):

(3) $$y_{t}^{\text{forward}}=Ch_{t}^{\text{forward}}$$

(4) $$y_{t}^{\text{backward}}=C_{b}h_{t}^{\text{backward}}$$

Here, *y_t_*denotes the forward output at time step *t*, and *C* represents the modality-specific output parameter.

Notably, depressive behaviors often exhibit asymmetric cross-modal temporal correlations between facial cues and verbal features. For instance, in individuals with subclinical depression, prosodic “pauses” show distinct time-locked associations with facial-related head postures such as “gaze straight ahead,” whereas in healthy individuals, vocal emotion “delight” is strongly synchronized with the facial expression “smile” (27). This reflects a fundamental temporal asymmetry in cross-modal synchronization. To address this challenge, a cross-modal attention mechanism is proposed to explicitly capture such temporal shift correlations (28, 29). The mathematical formulation (Equation 5) of the proposed mechanism is expressed as:

(5) $$\text{Attn}(Q,\;K,\;V)=\text{Softmax}(\frac{QK^{T}}{\sqrt{d_{k}}})V$$

This approach compensates for the limitations of conventional concatenation-based fusion in state space models (SSMs) by enabling refined alignment between modalities. Specifically, this attention mechanism models pairwise inter-modal relationships to generate two types of aligned features:

1. Audio-to-video aligned features: Audio queries interact with video keys/values to detect vocal cues that precede visual responses.
2. Video-to-audio aligned features: Visual queries probe audio contexts to identify delayed behavioral feedback.

#### 3.2.2 Multi-scale gated parallel fusion

In the detection of depression-related behavioral patterns, gradual degradation phenomena are frequently observed, which can be categorized as follows:

- Long-term behavioral attenuation: Progressive shortening of smile duration during conversation (11, 30);
- Periodic abnormalities: Significant decline in vocal energy fluctuation amplitude (31, 32);
- Micro-expression mutations: Abnormal increase in eye-closing action frequency (33, 34)

To address these multi-scale temporal pathological characteristics, a novel module integrating a multi-scale temporal feature pyramid with dynamic gating mechanisms is proposed for cross-granularity pathological feature extraction.

**Figure 5:**
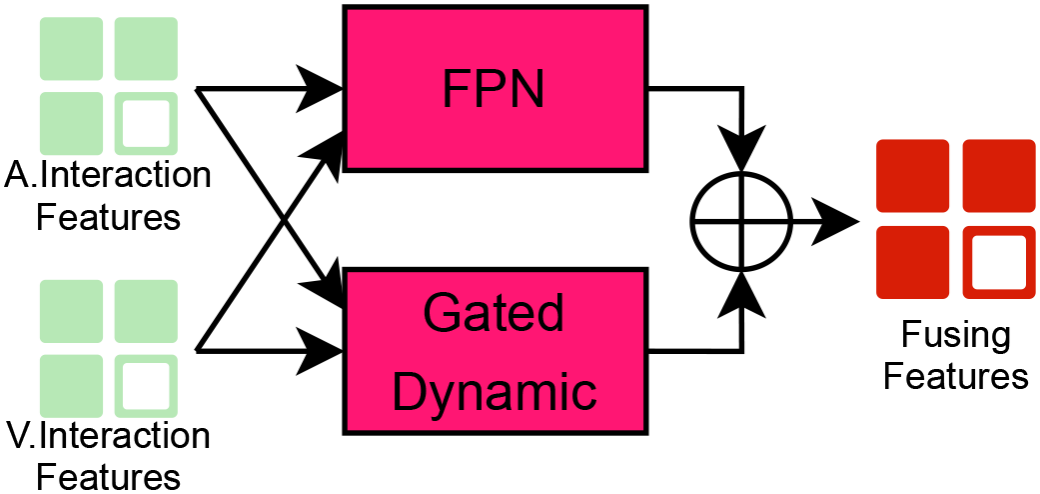
Multi-Scale Feature Fusion Pyramid (FPN) and Gating Mechanism.

As illustrated in **Figure 5**, the proposed architecture consists of two parallel branches:

1. Temporal Feature Pyramid Network (FPN): This branch performs hierarchical temporal abstraction through dilated convolutions and pyramid pooling.
2. Gated Dynamic Fusion: This branch performs environment-adaptive weighting through a gated self-adaptive mechanism.

This dual-path design facilitates the simultaneous modeling of millisecond-level micro-expression transients and minute-scale behavioral degradation trends, thereby effectively capturing the temporal heterogeneity inherent in depression manifestation (20, 22). The following two subsections will introduce the structures of these two pathways respectively.

##### 3.2.2.1 FPN

**Figure 6:**
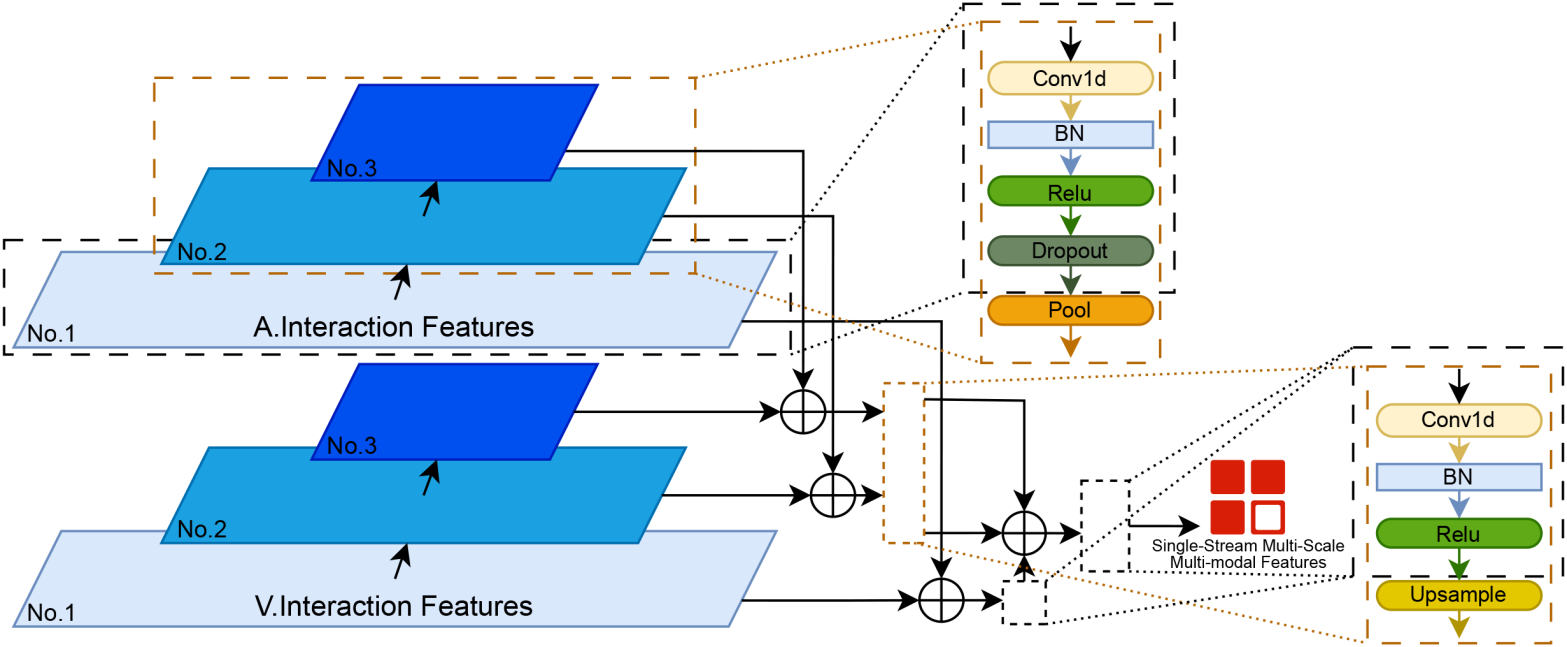
Structure of the feature pyramid network (FPN) for multimodal features.

The Feature Pyramid Network (FPN) employs hierarchical convolutional sampling to facilitate multi-modal feature extraction, constructing a three-tiered architecture that corresponds to three distinct scenarios observed in progressive degradation patterns. The structure of this multi-scale pyramidal layer is illustrated in **Figure 6**.

The first pyramid level (bottom level) is designed to detect transient events, capturing millisecond-level subtle facial movements (e.g., AU4 eyelid closure or brow furrowing). This enables the precise parsing of short-term behavioral dynamics. The formulation (Equation 6) for this process is given as follows:

(6) $$F_{fpm}^{1}=\text{Conv}1{\text{D}}_{3\times 1}(F_{in})\;(T\times D)$$

This level utilizes a one-dimensional convolution with a kernel size of 3 and a stride of 1 to process the input features (*F*_in_). This operation captures local temporal patterns while preserving a high temporal resolution, thereby constructing a foundational feature representation. Batch Normalization (BN) and Dropout layers are incorporated to standardize activations and mitigate overfitting, respectively, which aids in preserving local details. The output is a set of fine-grained features (*F_fpm_*^1^). Subsequently, features from congruent levels across different modalities are fused to produce aligned fine-grained multimodal representations.

The second level focuses on analyzing prosodic cycles (e.g., vocal energy fluctuations) to model the temporal dynamics inherent in speech prosody. The formulation (Equation 7) for this process is given as follows:

(7) $$F_{fpm}^{2}={\text{MaxPool}}_{2\times 1}(\text{Conv}1{\text{D}}_{3\times 1}(F_{fpm}^{1}))\;(T/2\times D)$$

Compared to the architecture of the previous stage, this stage incorporates an additional max-pooling operation with both the pooling window size and stride set to 2. This downsampling extracts medium-scale features (*F_fpm_*^2^) from the fine-grained features (*F_fpm_*^1^), effectively aggregating local information into a more global context. The cross-modal fusion strategy at this level extends the approach of the first level by incorporating upsampling to restore feature dimensions, thereby aligning medium-scale features for subsequent cross-level fusion.

The third level captures longitudinal behavioral trends (e.g., the decay of smile duration during conversations) to model evolving patterns over extended temporal contexts. The formulation (Equation 8) for this process is given as follows:

(8) $$F_{fpm}^{3}={\text{MaxPool}}_{2\times 1}(\text{Conv}1{\text{D}}_{3\times 1}(F_{fpm}^{2}))\;(T/4\times D)$$

This level employs the same structure as the second level, utilizing max−pooling to further downsample the features. This process extracts coarse-scale features $\left(F_{fpm}^{3}\right)$ from the medium-grained features $\left(F_{fpm}^{2}\right)$, further integrating information across receptive fields. The strategy for fusing cross-modal features at this level remains consistent with that of the second level.

Finally, the output at each layer $\left(F_{A.fpm}^{l}\right)$ is concatenated with its corresponding output from the other modality $\left(F_{V.fpm}^{l}\right)$. This yields three feature representations with varying sequence lengths. Subsequently, using the bottom-layer features as the reference for temporal resolution, the middle- and top-layer features are upsampled accordingly. All three sets of features are then concatenated to produce a comprehensive representation $\left(F_{fused}\right)$, which integrates complementary information from multiple modalities along with their respective multi-scale characteristics. The mathematical formulation (Equation 9) of the final fusion process is given as follows:

(9) $$F_{fused}=\text{Upsample}({\text{Conv}}_{1\times 1}({⊕}_{l=1}^{3}(F_{A.fpm}^{l}⊕F_{V.fpm}^{l})))$$

##### 3.2.2.2 Gated dynamic fusion

**Figure 7:**
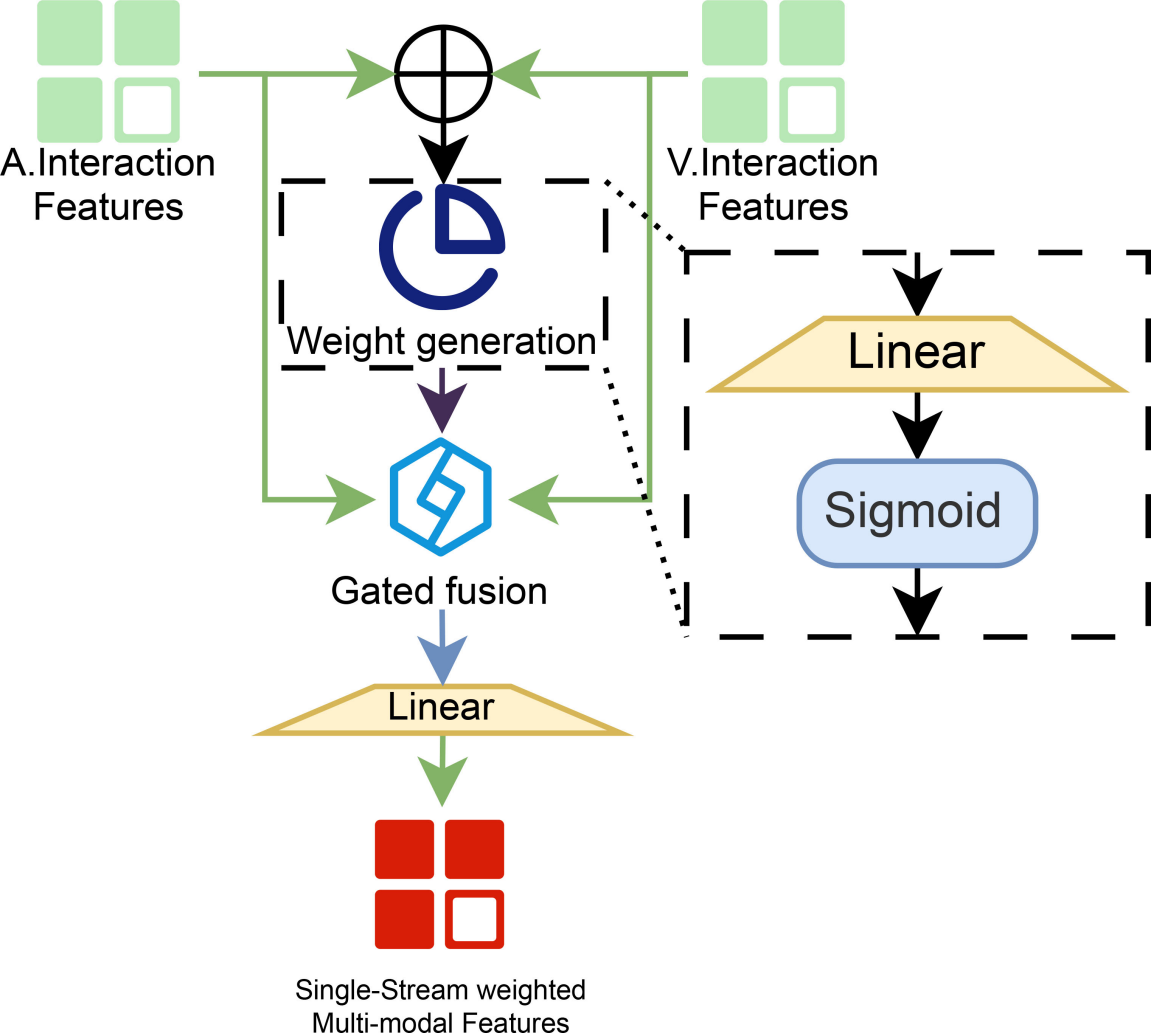
Workflow of the gated dynamic fusion module.

The architecture of the gated dynamic fusion module is illustrated in **Figure 7**.

The gated dynamic fusion module accepts two parallel streams of input features: the audio interaction features and the video interaction features. These two modalities are first concatenated along the feature dimension and passed into a weight-generation sub-module. This sub-module consists of a linear layer followed by a sigmoid activation, which produces a set of adaptive fusion weights that vary with the input context. The derived weight vector is then combined with each modality stream via a dedicated gated fusion unit, where features from the audio and visual branches are modulated by the corresponding gating values. The fusion weight *γ* is derived as follows (Equation 10):

(10) $$\gamma =\sigma (W_{g}\cdot [{\text{Features}}_{\text{audio}}⊕{\text{Features}}_{\text{video}}])$$

Finally, the gated-fused representation undergoes a linear projection to integrate and refine the multi-modal information, yielding a consolidated single-stream output termed the weighted multi-modal features.

Following parallel fusion, the two resultant features undergo dimensional concatenation and alignment, ultimately producing complementarity-enhanced features that facilitate temporal multi-granularity modeling.

#### 3.2.3 Multimodal enhanced mamba

**Figure 8:**
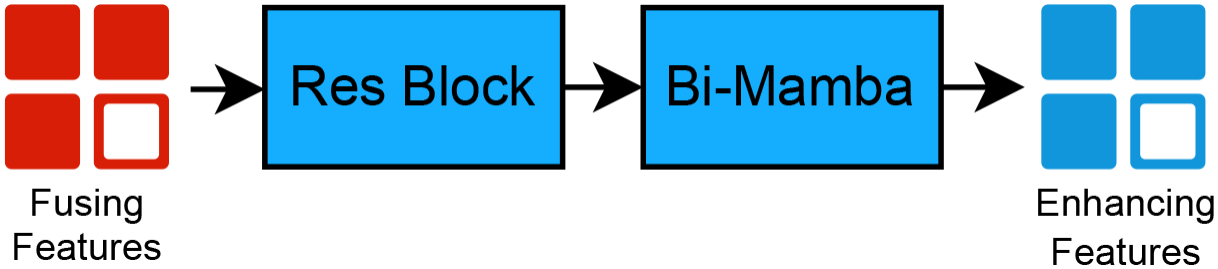
Feature enhancement process via residual blocks and bi-mamba.

To further enhance the fused multimodal features while preserving the inherent advantages of Mamba in terms of computational efficiency and memory footprint, our multimodal enhancement module adopts an architectural design rationale analogous to that employed in the DepMamba model (25). In the original design, the state matrices within the forward and backward State Space Model (SSM) modules are updated through joint interactive learning from separate audio and video inputs; in this module, the state matrices of the bidirectional SSM modules are both driven and updated by a single cross-modal fused feature stream. These modules perform global context modeling at this stage to enhance features, with their architecture illustrated in **Figure 8**.

**Figure 9:**
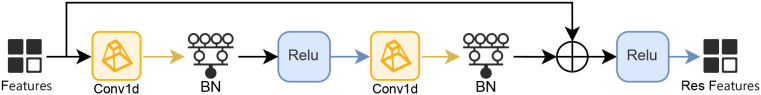
Schematic of the residual block (ResBlock).

The residual block structure within this module is consistent with that in the Multi-Scale Gated Parallel Fusion module, as shown in **Figure 9**. This residual architecture employs two stacked 1D convolutional layers for hierarchical feature extraction: the first layer captures local temporal patterns, while the second layer derives more abstract higher-order features from the previous layer’s output. Batch Normalization (BN) layers and Rectified Linear Unit (ReLU) activation are incorporated to stabilize training dynamics, enhance representational capacity, and mitigate issues such as vanishing gradients and temporal information loss.

After feature refinement and enhancement through the Multimodal Enhanced Mamba module, a linear classifier is employed for final discriminative classification, enabling depression diagnosis.

## 4 Results

All experiments were conducted on a hardware platform equipped with an AMD Ryzen 5–5600 CPU, 64-bit Ubuntu 24.04 LTS operating system, 64GB DDR4 RAM, and an NVIDIA RTX 2080Ti GPU with 22GB GDDR6 VRAM. The software environment utilized Python 3.10 with the PyTorch 2.1 framework accelerated by CUDA 11.7.

The Adam optimizer was employed with an initial learning rate of 8e-5 and L2 weight decay (*λ* = 0.001) to mitigate overfitting. Dataset-specific training protocols were implemented: 16 epochs for D-Vlog and 12 epochs for LMVD, corresponding to their respective temporal complexities. The feature extraction module employed 1×1 convolutional kernels with 256 output channels, while the hidden dimension of recurrent units was uniformly set to 256 across all architectures. State dimensions were configured to 16 for D-Vlog and 12 for LMVD to accommodate their distinct temporal patterns. Due to hardware constraints, the number of cross-modal attention heads was limited to 2 for computational efficiency.

To rigorously evaluate model performance, a comprehensive assessment was conducted using four established classification metrics: accuracy, precision, recall, Specificity, and F1-score. These metrics are defined as follows:


$$Accuracy=\frac{TP+TN}{TP+TN+FP+FN}$$

$$Precision=\frac{TP}{TP+FP}$$

$$Recall=\frac{TP}{TP+FN}$$

$$Specificity=\frac{TN}{TN+FP}$$

$$F_{1}=2\times \frac{Precision\times Recall}{Precision+Recall}$$

### 4.1 Comparison results

To validate the effectiveness of our proposed model, we benchmarked it against widely recognized multimodal depression detection methods on two large-scale datasets (D-Vlog and LMVD), with statistical significance tests, fine-grained classification analysis, and computational efficiency comparisons to verify the robustness of our performance improvements.

#### 4.1.1 Comparison on D-Vlog and LMVD

As demonstrated in **Tables 3** and **4**, our proposed model exhibits superior performance in both accuracy and F1-score metrics. On the D-Vlog dataset, it achieves an accuracy of 70.91%, representing a significant improvement of 3.0 percentage points over the second-best performing model. The F1-score reaches 77.13%, exceeding the runner-up model by 0.69 percentage points. The model achieves an aggregate score of 71.37% across all metrics, outperforming the second-best method by 2.52 percentage points. Evaluations on the LMVD dataset further confirm the model’s effectiveness and robustness. The accuracy reaches 74.68%, demonstrating a 2.55 percentage point advantage over the second-best approach. While maintaining precision at 73.87%, our model preserves a 2.72 percentage point lead. The F1-score reaches 74.90%,

**Table 3:** Performance comparison on the D-Vlog.

Model	Accuracy (%)	Precision (%)	Recall (%)	Specificity (%)	F1(%)	Avg (%)
STFT (35)	62.17	63.77	77.28	41.32	69.91	62.89
TAMFN (36)	66.95	67.89	82.68	43.71	74.81	67.21
DepDetector (23)	62.87	64.52	84.82	29.86	72.54	62.92
TFN (37)	67.12	**72**.**38**	70.19	62.86	71.23	68.76
DepMamba (25)	68.87	68.19	**86**.**99**	43.76	76.44	68.85
KNN (38)	61.38	61.41	85.64	29.88	71.47	61.96
TBN (39)	63.23	69.98	65.35	**59**.**22**	67.11	64.98
Bi-LSTM (40)	64.47	67.68	75.34	48.09	71.10	65.34
ASYM (Ours)	**70**.**91**	70.95	84.55	53.27	**77**.**13**	**71**.**37**

Bold values indicate the best performance in each column.

**Table 4:** Performance comparison on the LMVD.

Model	Accuracy (%)	Precision (%)	Recall (%)	Specificity (%)	F1 (%)	Avg (%)
STFT (35)	67.69	69.21	64.11	70.29	66.21	67.5
TAMFN (36)	70.46	71.12	68.79	71.13	69.83	70.27
DepDetector (23)	61.93	60.36	72.16	51.45	65.08	62.2
TFN (37)	63.88	64.12	62.62	65.13	63.33	63.82
DepMamba (25)	72.13	70.18	**76.56**	67.74	73.2	71.96
KNN (38)	56.83	57.43	50.92	62.67	53.92	56.35
TBN (39)	67.93	67.11	70.33	65.53	68.49	67.88
Bi-LSTM (40)	66.84	65.83	70.32	63.45	67.81	66.85
ASYM (Ours)	**74.68**	**73.87**	76.19	**73.28**	**74.9**	**74.59**

Bold values indicate the best performance in each column.

exceeding the second-best model by 1.70 percentage points.The model yields an overall average score of 74.59% across all metrics, outperforming the second-best method by a notable margin of 2.63 percentage points. These consistent improvements across both datasets substantiate the model’s generalizability and detection capabilities in multimodal depression analysis.

#### 4.1.2 Statistical significance verification

To validate the effectiveness of our proposed model, we conducted comparisons with mainstream multimodal depression detection methods on two large-scale datasets (D-Vlog and LMVD) via 5-fold cross-validation, with results presented in **Tables 5** and **6**. On the D-Vlog dataset, our model achieved an accuracy of 71.35% and an F1-score of 77.36%, outperforming the second-best performing model DepMamba by 2.60 and 0.99 percentage points in accuracy and F1-score, respectively. On the LMVD dataset, the proposed method reached a top accuracy of 74.32% and an F1-score of 74.59%, with a 2.19 percentage point improvement in accuracy and a 1.30 percentage point gain in F1-score compared with the runner-up model. We also performed one-sided Wilcoxon signed-rank tests to verify the statistical significance of the performance improvements, and the results confirmed that the superiority of our model.

**Table 5:** Performance comparison on the D-Vlog via 5-Fold cross-validation.

Model	Accuracy (%)	Precision (%)	Recall (%)	Specificity (%)	F1 (%)
STFT **	61.98 ± 1.2	64.18 ± 1.7	77.48 ± 4.4	40.74 ± 1.5	70.21 ± 1.9
TAMFN*	66.15 ± 1.1	66.67 ± 1.8	82.88 ± 1.1	43.21 ± 0.9	73.89 ± 1.6
DepDetector *	61.46 ± 2.1	62.25 ± 2.4	84.68 ± 5.8	29.63 ± 1.6	71.75 ± 3.4
TFN **	67.19 ± 0.7	72.22 ± 1.7	70.27 ± 3.2	62.96 ± 2.2	71.22 ± 2.1
DepMamba *	68.75 ± 2.2	67.83 ± 1.8	87.39 ± 0.9	43.21 ± 0.7	76.37 ± 1.9
KNN **	61.98 ± 1.1	62.50 ± 1.2	85.59 ± 4.3	29.63 ± 1.9	72.24 ± 1.6
TBN *	63.02 ± 2.1	68.87 ± 2.4	65.77 ± 4.8	59.26 ± 2.6	67.28 ± 2.4
Bi-LSTM *	64.06 ± 0.4	66.67 ± 1.9	75.68 ± 3.2	48.15 ± 2.3	70.88 ± 1.3
ASYM(Our)	71.35 ± 0.9	71.21 ± 1.1	84.68 ± 1.2	53.09 ± 1.4	77.36 ± 0.8

All values are presented as mean ± standard deviation. Statistical significance is tested by one-sided Wilcoxon signed-rank test based on paired 5-fold cross-validation results, compared with the ASYM. (*P < 0.05, **P < 0.01).

**Table 6:** Performance comparison on the LMVD via 5-Fold cross-validation.

Model	Accuracy (%)	Precision (%)	Recall (%)	Specificity (%)	F1 (%)
STFT *	67.21 ± 1.1	68.24 ± 2.8	63.74 ± 4.5	70.65 ± 1.8	65.92 ± 3.2
TAMFN *	69.95 ± 0.6	70.26 ± 2.6	69.23 ± 2.3	70.65 ± 0.9	69.61 ± 2.4
DepDetector **	61.75 ± 2.7	59.46 ± 3.4	72.53 ± 4.8	51.09 ± 1.6	65.34 ± 4.4
TFN **	63.93 ± 1.9	64.04 ± 2.1	62.64 ± 0.9	65.22 ± 2.2	63.33 ± 1.4
DepMamba *	72.13 ± 1.2	70.13 ± 1.8	76.92 ± 2.5	67.39 ± 0.7	73.29 ± 2.3
KNN **	56.83 ± 1.6	57.50 ± 1.6	50.55 ± 4.3	63.04 ± 1.4	53.81 ± 2.7
TBN **	67.76 ± 2.1	66.67 ± 2.9	70.33 ± 3.8	65.22 ± 0.7	68.45 ± 3.4
Bi-LSTM **	66.67 ± 1.7	65.31 ± 3.4	70.33 ± 4.2	63.04 ± 2.4	67.72 ± 3.6
ASYM(Our)	74.32 ± 1.3	73.40 ± 1.4	75.82 ± 0.9	72.83 ± 1.7	74.59 ± 1.5

All values are presented as mean ± standard deviation. Statistical significance is tested by one-sided Wilcoxon signed-rank test based on paired 5-fold cross-validation results, compared with the ASYM. (*P < 0.05, **P < 0.01).

over all baseline methods is statistically significant (p*<*0.05 or p*<*0.01). These experimental results fully demonstrate the superior performance of the ASYM model in multimodal depression detection.

#### 4.1.3 Classification performance analysis

**Figure 10:**
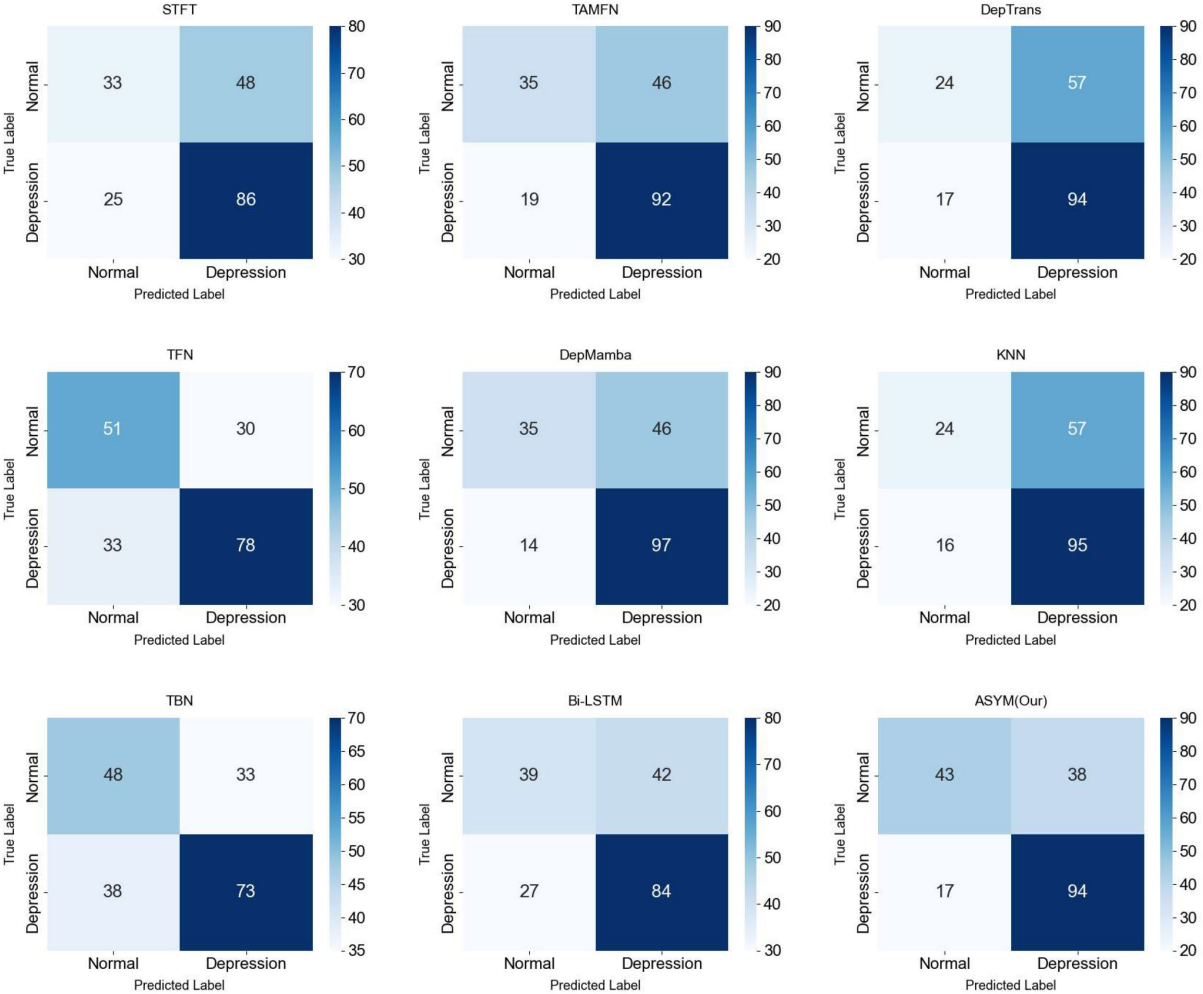
Confusion matrices on the D-Vlog.

**Figure 11:**
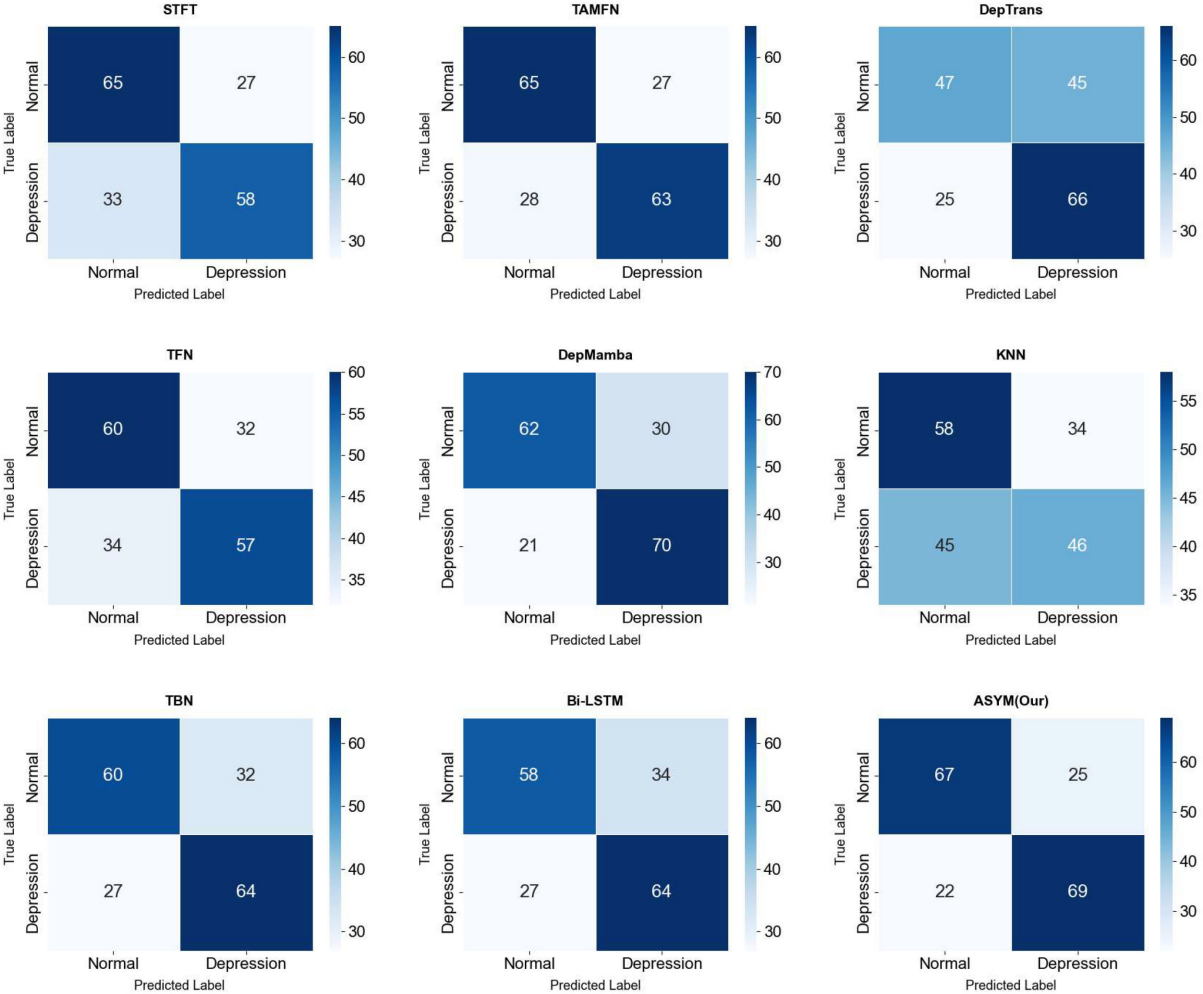
Confusion matrices on the LMVD.

**Figure 12:**
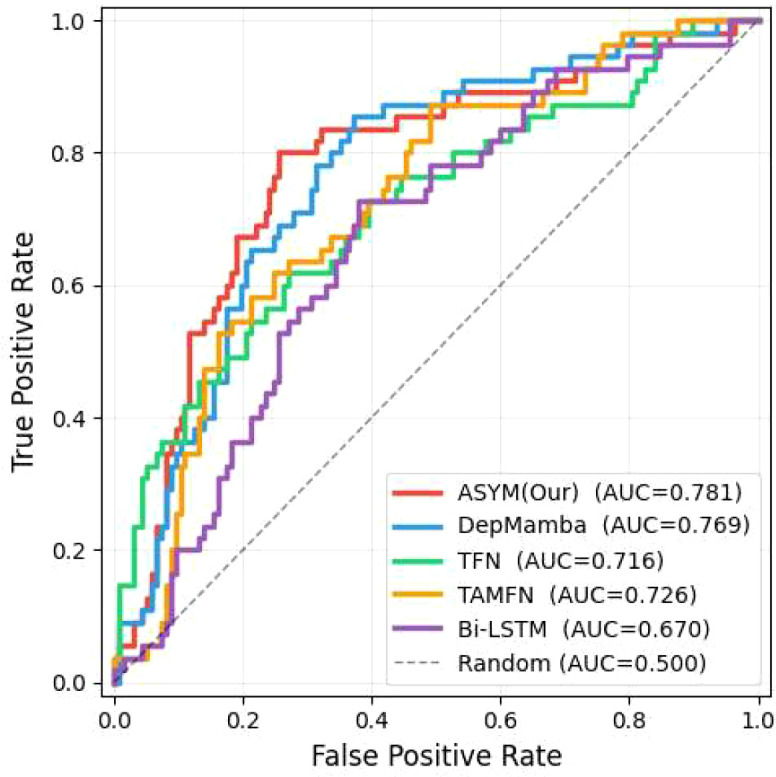
ROC curves and AUC values on the D-Vlog.

**Figure 13:**
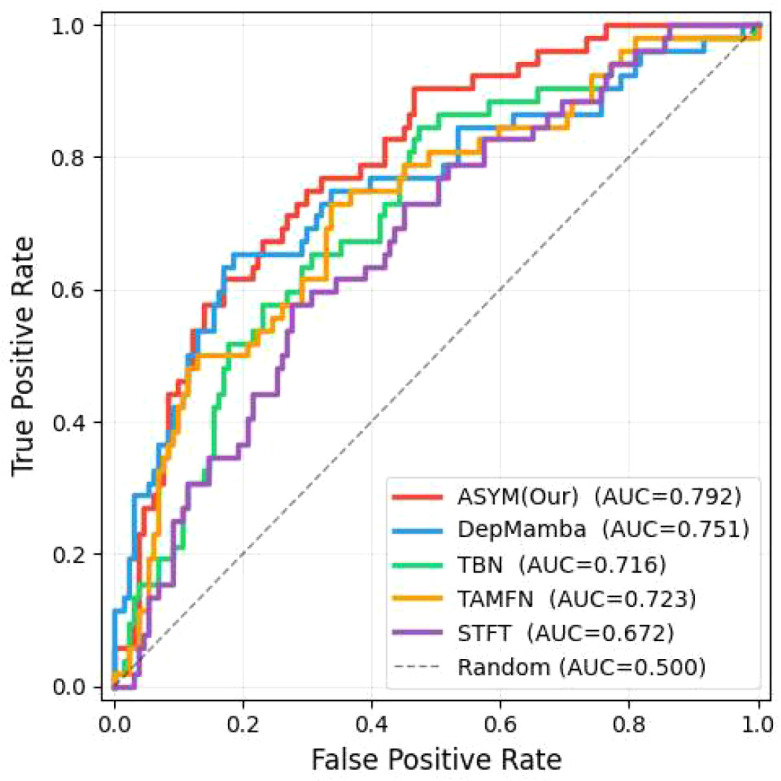
ROC curves and AUC values on the LMVD.

**Figures 10**-**13** provide comprehensive visual performance validation of our proposed ASYM model against baseline methods on the D-Vlog and LMVD datasets: specifically, **Figures 10** and **11** show the confusion matrices (rows: ground truth labels; columns: predicted labels), where our model achieves the largest number of correctly classified samples, while **Figures 12** and **13** present the Receiver Operating Characteristic (ROC) curves, with our ASYM model reaching the highest Area Under the Curve (AUC) values (0.781 on D-Vlog, 0.792 on LMVD) among all competing baselines, further corroborating its superior overall classification capability for multimodal depression detection tasks.

#### 4.1.4 Efficiency comparison

**Figure 14:**
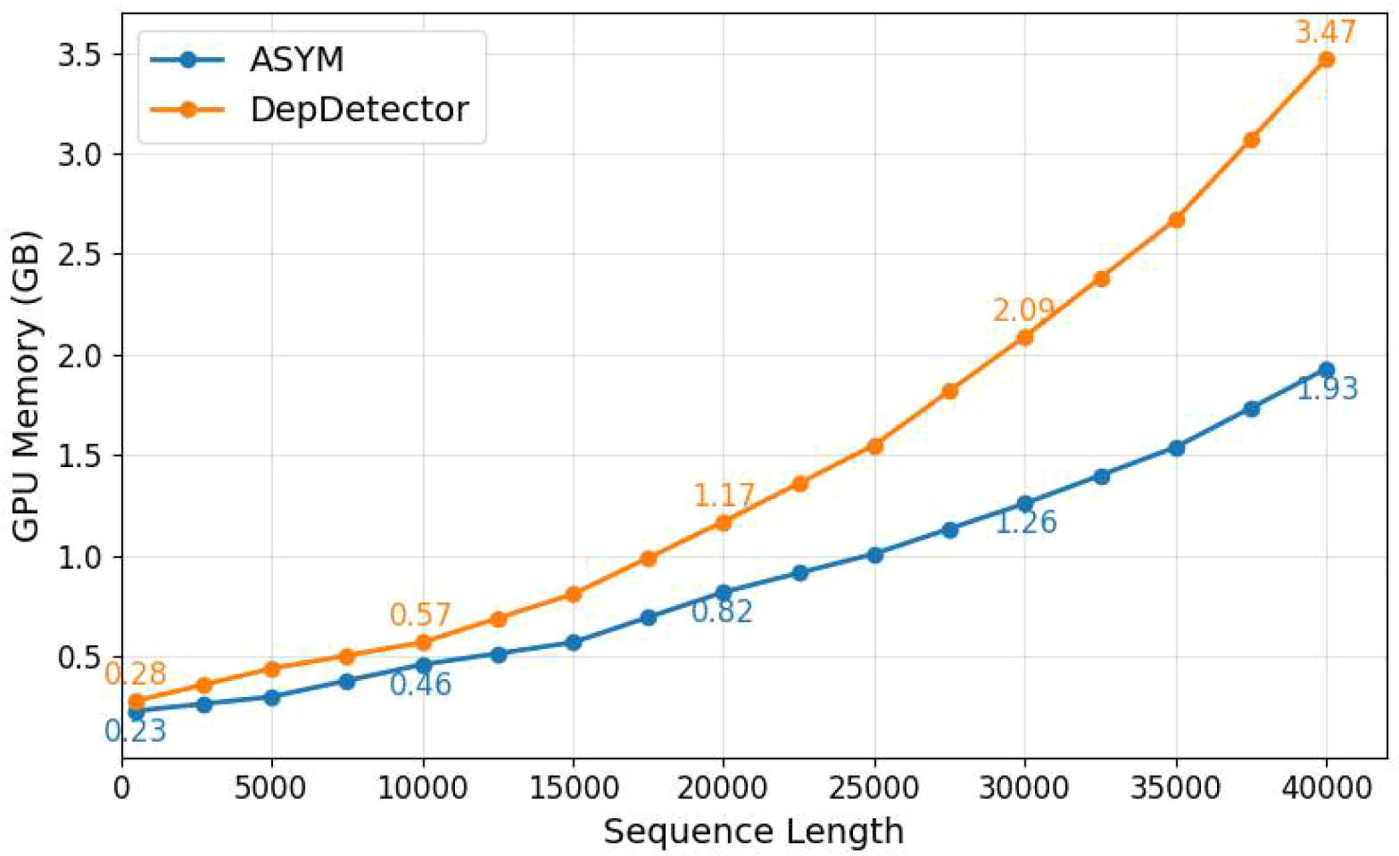
GPU memory comparison.

As demonstrated in **Table 7**, the proposed ASYM framework exhibits superior parameter efficiency, containing only 0.87M parameters compared to DepDetector’s 1.04M, which represents a 12.6% reduction in model size. Furthermore, ASYM consistently requires lower computational cost (FLOPs) than DepDetector across all tested sequence lengths. The memory efficiency is corroborated by **Figure 14**, which shows that while the GPU memory consumption of both models scales with sequence length, ASYM maintains a significantly lower memory footprint. For instance, at a sequence length of 40,000, ASYM consumes merely 1.93 GB, substantially less than the 3.47 GB required by DepDetector, achieving a memory saving of approximately 44.4%.

**Table 7:** Efficiency comparisons at different sequence length between ASYM and DepDetector.

Method	Params (M)	FLOPs (G)				
		0.5k	1k	10k	20k	50k
DepDetector	1.04	0.14	0.27	2.79	5.59	13.87
ASYM	0.87	0.09	0.16	1.28	2.57	6.45

### 4.2 Cross-modal attention visualization analysis

**Figure 15:**
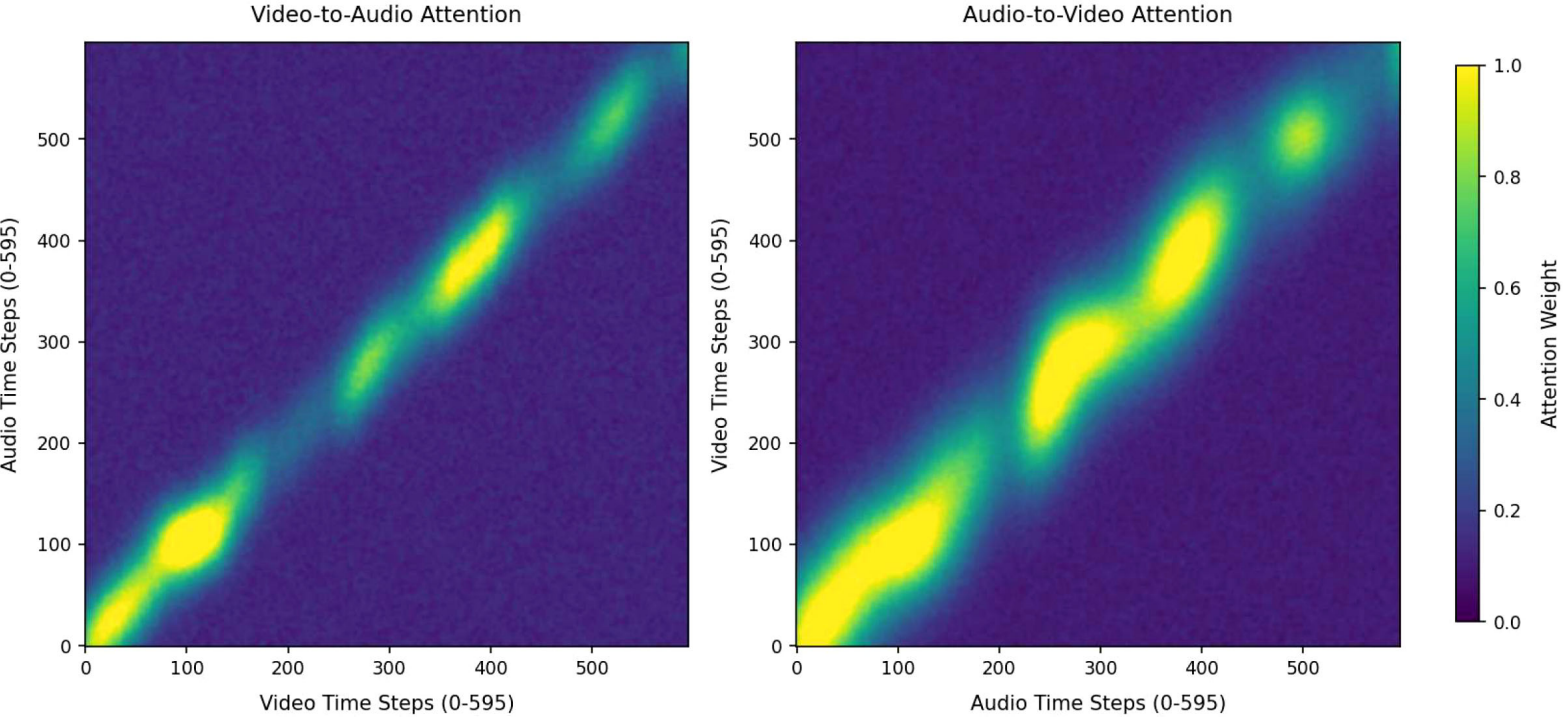
Cross-modal attention heatmaps on the D-Vlog.

**Figure 16:**
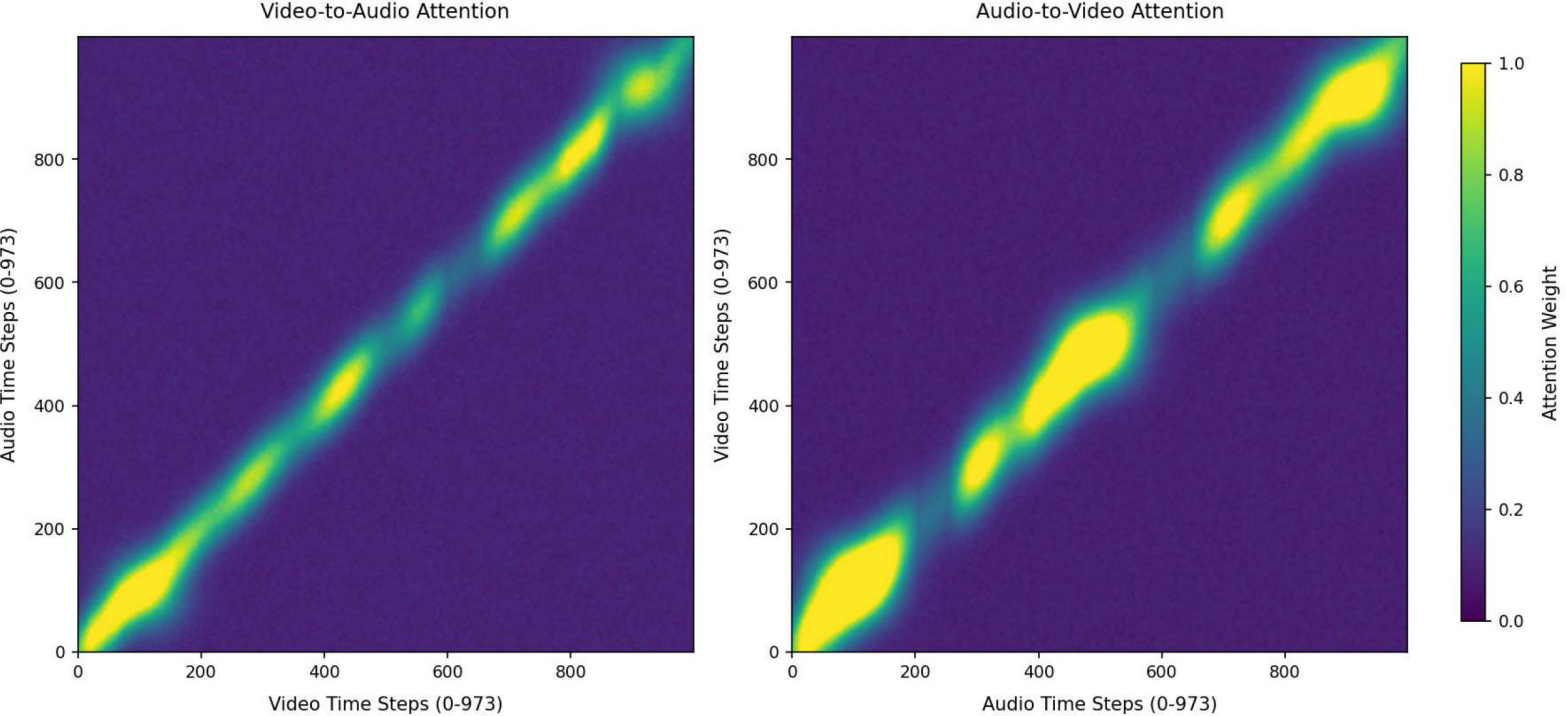
Cross-modal attention heatmaps on the LMVD.

As illustrated in **Figures 15** and **Figures 16**, for both the D-Vlog and LMVD datasets, the high-attention-weight regions of bidirectional cross-modal attention (both Video-to-Audio and Audio-to-Video directions) are concentrated along the diagonal of the heatmaps. This observation demonstrates the inherent temporal alignment between audio and video modalities during cross-modal interaction. Meanwhile, noticeable fluctuations and undulations are observed in the high-weight regions along the diagonal, indicating that the cross-modal interaction is not strictly aligned at the individual time-step level. This temporal fluctuation pattern directly aligns with our earlier finding that depressive behaviors show asymmetric cross-modal temporal correlations between video-derived facial cues and audio-derived verbal features.

**Figure 17:**
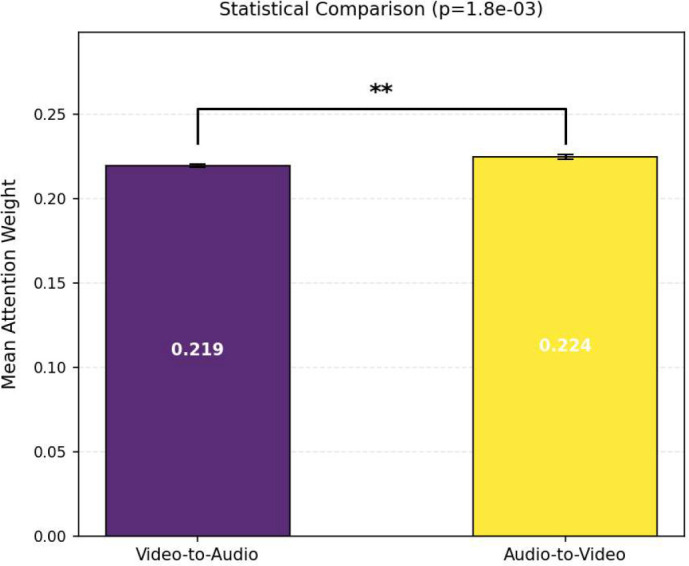
Statistical comparison of cross-modal attention weights on the D-Vlog. The double asterisk (**) indicates a statistically significant difference between the two conditions (p<0.01).

**Figure 18:**
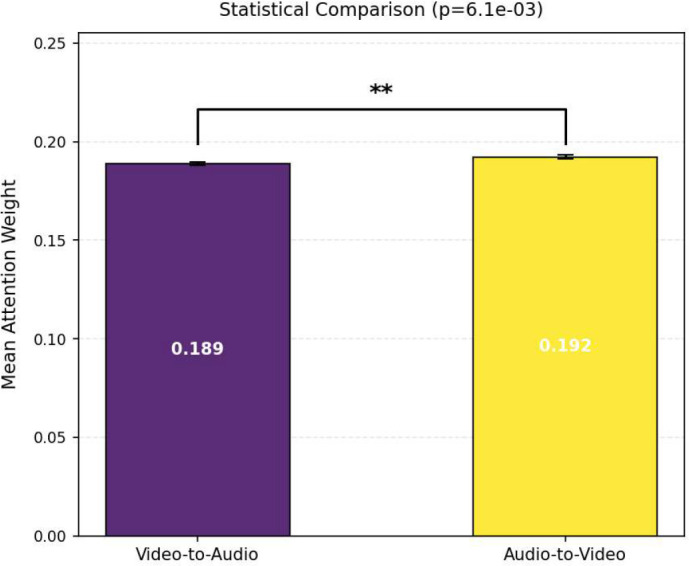
Statistical comparison of cross-modal attention weights on the LMVD. The double asterisk (**) indicates a statistically significant difference between the two conditions (p<0.01).

Furthermore, as shown in **Figures 17** and **Figure 18**, the mean attention weights of the Audio-to-Video and Video-to-Audio directions present a statistically extremely significant difference (p *<* 0.01) on both the D-Vlog and LMVD datasets, with the attention intensity of the Audio-to-Video direction being significantly.

higher. Consistent with this finding, the unimodal ablation results in **Tables 8** and **9** reveal that the audio-only model achieves better detection performance than the video-only model on both datasets. This quantitative performance gap further corroborates the visual observation in **Figures 15** and **Figure 16** that the Audio-to-Video attention presents higher intensity and wider coverage of high-weight regions.

**Table 8:** D-Vlog ablation study: audio and video.

Audio	Video	Accuracy (%)	Precision (%)	Recall (%)	Specificity (%)	F1(%)
✓	ઞ	59.68	62.83	77.08	34.81	69.23
ઞ	✓	67.35	65.14	80.74	52.69	72.11
✓	✓	**70**.**91**	**70**.**95**	**84**.**55**	**53**.**27**	**77**.**13**

Bold values indicate the best performance in each column.

**Table 9:** LMVD ablation study: audio and video.

Audio	Video	Accuracy (%)	Precision (%)	Recall (%)	Specificity (%)	F1(%)
✓	ઞ	69.37	63.44	71.28	67.88	67.13
ઞ	✓	71.79	70.48	72.59	71.03	71.52
✓	✓	**74**.**68**	**73**.**87**	**76**.**19**	**73**.**28**	**74**.**90**

Bold values indicate the best performance in each column.

Collectively, these experimental results demonstrate that bidirectional cross-modal attention interaction has complementary characteristics, and audio information serves as the core guiding modality for cross-modal depression detection.

### 4.3 Ablation study

To thoroughly evaluate the effectiveness of the proposed method, we conducted a systematic ablation study focusing on two key aspects: input modalities and model architecture. Comparative experiments were performed accordingly.

#### 4.3.1 Ablation on input modalities

As detailed in **Tables 8** and **9**, replacing the full multimodal input with either unimodal (audio-only or video-only) setup leads to a consistent performance drop across both datasets. Notably, the audio-only model exhibits the most severe degradation, particularly on the D-Vlog dataset.This result demonstrates that our multimodal learning strategy effectively integrates complementary information from both modalities.

#### 4.3.2 Ablation on baseline model

To validate whether the selected baseline model Bi-Mamba outperforms the conventional unidirectional Mamba in long-sequence modeling and contextual understanding tasks, we conducted an ablation study within the ASYM framework, focusing exclusively on the baseline models across the D-Vlog and LMVD datasets. As shown in **Tables 10** and **11**, ASYM with Bi-Mamba as the baseline achieves superior performance in Accuracy, Precision, Recall, and F1-score on both datasets, as well as in Specificity on the LMVD dataset, compared to ASYM with Mamba as the baseline. The only exception occurs in Specificity on the D-Vlog dataset, where the Bi-Mamba variant falls slightly behind. Overall, these results confirm the stronger contextual modeling capability of Bi-Mamba in the given tasks.

**Table 10:** Baseline model performance: mamba vs. bi-mam on the D-Vlog.

Model	Accuracy (%)	Precision (%)	Recall (%)	Specificity (%)	F1(%)
Mamba	67.63	66.53	77.67	**55.44**	71.67
Bi-Mamba	**70.91**	**70.95**	**84.55**	53.27	**77.13**

Bold values indicate the best performance in each column.

**Table 11:** Baseline model performance: mamba vs. bi-mamba on the LMVD.

Model	Accuracy (%)	Precision (%)	Recall (%)	Specificity (%)	F1 (%)
Mamba	65.78	68.37	74.62	53.98	71.36
Bi-Mamba	**74.68**	**73.87**	**76.19**	**73.28**	**74.90**

Bold values indicate the best performance in each column.

#### 4.3.3 Ablation on model architecture

Interaction Modules: Ablating either the Bi-Mamba or the Cross-Modal Attention (CMA) module results in a significant decline across most metrics, as shown in **Tables 12** and **13**. An interesting observation on the D-Vlog dataset is that the model with only one interaction module (either Bi-Mamba or CMA) achieves a higher Specificity than the full model. This suggests that the synergistic use of both Bi-Mamba and CMA enables the model to more accurately identify positive samples (higher Recall and F1), albeit with a slight trade-off in specificity.

**Table 12:** D-Vlog ablation study: CM attention.

CM attention	Accuracy (%)	Precision (%)	Recall (%)	Specificity (%)	F1 (%)
ઞ	66.34	63.58	73.98	**57**.**94**	68.39
✓	**70**.**91**	**70**.**95**	**84**.**55**	53.27	**77**.**13**

Bold values indicate the best performance in each column.

**Table 13:** LMVD ablation study: CM attention.

CM attention	Accuracy (%)	Precision (%)	Recall (%)	Specificity (%)	F1 (%)
ઞ	69.82	67.78	70.83	68.89	69.27
✓	**74**.**68**	**73**.**87**	**76**.**19**	**73**.**28**	**74**.**90**

Bold values indicate the best performance in each column.

Fusion Architecture: The importance of our dual-path fusion design is validated in **Tables 14** and **15**. Removing either the Feature Pyramid Network (FPN) or the Gated Dynamic (GD) fusion module degrades performance. Specifically, using FPN alone yields a higher Specificity (55.98% vs. 53.27% on D-Vlog).

**Table 14:** D-Vlog ablation study: FPN and gated dynamic (GD).

FPN	GD	Accuracy (%)	Precision (%)	Recall (%)	Specificity (%)	F1 (%)
✓	ઞ	69.20	68.01	79.87	**55**.**98**	73.46
ઞ	✓	68.31	67.61	82.52	50.53	74.32
ઞ	ઞ	63.73	65.49	77.28	45.64	70.90
✓	✓	**70**.**91**	**70**.**95**	**84**.**55**	53.27	**77**.**13**

Bold values indicate the best performance in each column.

**Table 15:** LMVD ablation study: FPN and gated dynamic (GD).

FPN	GD	Accuracy (%)	Precision (%)	Recall (%)	Specificity (%)	F1 (%)
✓	ઞ	72.48	71.44	73.35	71.64	72.38
ઞ	✓	70.82	69.36	75.58	65.97	72.34
ઞ	ઞ	70.20	68.04	71.83	68.69	69.88
✓	✓	**74**.**68**	**73**.**87**	**76**.**19**	**73**.**28**	**74**.**90**

Bold values indicate the best performance in each column.

but poorer overall performance (F1: 73.46% vs. 77.13%). This indicates that FPN alone biases the model towards conservative negative-sample identification, while integrating GD is essential for achieving a balanced and superior fusion for positive sample recognition.

Feature Enhancement Module: Finally, removing the Enhanced Mamba performance decline across both datasets, as detailed in **Tables 16** and **17**, validating its effectiveness in 388 refining the fused multimodal feature representations.

**Table 16:** D-Vlog ablation study: enhanced mamba (En-Mamba).

En-mamba	Accuracy (%)	Precision (%)	Recall (%)	Specificity (%)	F1 (%)
ઞ	64.12	67.98	76.31	45.71	71.90
✓	**70**.**91**	**70**.**95**	**84**.**55**	53.27	**77**.**13**

Bold values indicate the best performance in each column.

**Table 17:** LMVD ablation study: enhanced mamba (En-Mamba).

En-mamba	Accuracy (%)	Precision (%)	Recall (%)	Specificity (%)	F1 (%)
ઞ	71.98	67.05	72.45	71.61	69.65
✓	**74**.**68**	**73**.**87**	**76**.**19**	**73**.**28**	**74**.**90**

Bold values indicate the best performance in each column.

In summary, the ablation study demonstrates that each proposed component—multimodal input, the Bi-Mamba and CMA interaction duo, the FPN+GD fusion architecture, and the Enhanced Mamba refinement—plays a critical and non-redundant role in achieving the final robust performance.

### 4.4 Calibration analysis

To evaluate the reliability of the prediction probabilities generated by the ASYM model, we conducted a qualitative analysis of its calibration characteristics. We obtained the model’s prediction probabilities on the validation set and divided them into 10 equal-width intervals (i.e., 0.0–0.1, 0.1–0.2,…, 0.9–1.0), and the comparison between the average predicted probability and the actual observed positive rate within each interval demonstrated excellent consistency across all bins. Specifically, on the D-Vlog dataset, the average predicted probability and actual positive rate were 0.87 and 0.83 for high-confidence samples (prediction probability *>* 0.8), and 0.54 and 0.48 for the medium-confidence interval (0.4–0.6), respectively; on the LMVD dataset, these values were 0.84 and 0.81 for high-confidence samples, and 0.58 and 0.53 for the medium-confidence interval, respectively. No systematic bias was observed where the predicted probabilities were consistently higher or lower than the true incidence rates, demonstrating that the probability outputs of the ASYM model are well-calibrated and thus provide a reliable reference for the assessment of depression risk.

## 5 Conclusions and future work

This study presents ASYM, an innovative multimodal depression recognition framework that synergistically integrates dynamic gated fusion with hierarchical feature pyramid architecture. Our methodology fundamentally advances pathological pattern characterization through two key innovations:

1. A dual-path fusion architecture that establishes comprehensive temporal representations spanning millisecond-level micro-expression transients to longitudinal behavioral degradation trends, effectively addressing detection challenges including chronic behavioral attenuation, periodic abnormalities, and micro-expression mutations;
2. A novel Mamba-enhanced architecture with cross-modal attention synergy that overcomes traditional limitations in long-range temporal modeling efficiency while achieving deep multimodal feature integration. The experimental data confirm the strong performance of the proposed method, as it achieves superior results across a comprehensive set of evaluation metrics. The introduced cross-modal attention mechanism and multi-scale fusion strategy provide novel technical pathways for computational psychiatry, particularly in addressing temporal heterogeneity and multimodal asynchrony inherent in depression manifestation. Future work will focus on clinical validation across diverse demographic groups and extension to other neuropsychiatric disorders.

## Data Availability

Publicly available datasets were analyzed in this study. This data can be found here: 1.D-Vlog Dataset: Available at: https://sites.google.com/view/jeewoo-yoon/dataset2.LMVD Dataset: Available at: https://github.com/helang818/LMVD.
